# Supplementary figures and images for: Revealing spatio-temporal dynamics with long-term trypanosomatid live-cell imaging
Source: PLoS Pathog. 2022 Jan 18;18(1):e1010218. doi: 10.1371/journal.ppat.1010218 (PMC8797261; doi:10.1371/journal.ppat.1010218)

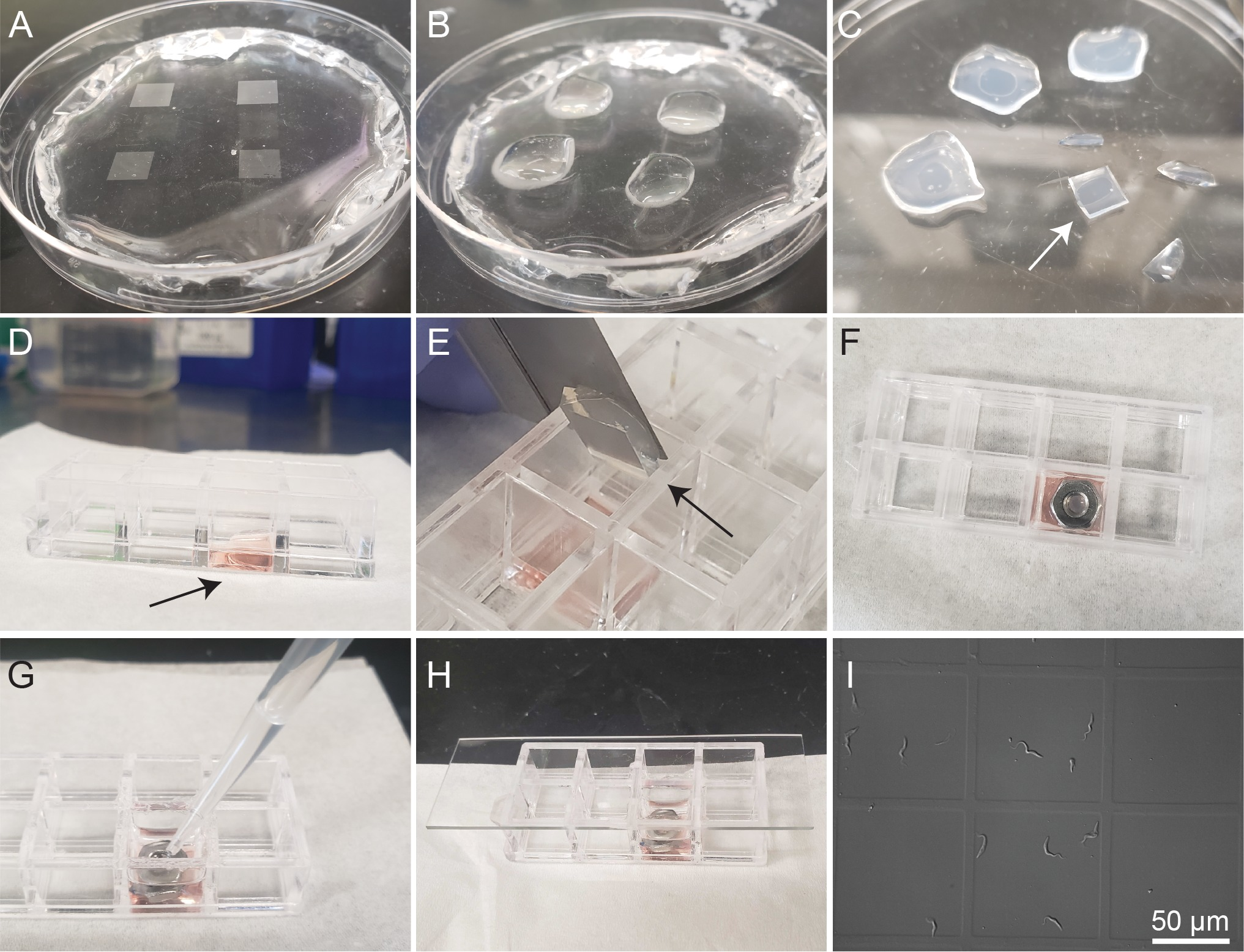

Supplement: S1 Fig — A. Representative image of the PDMS mold used to cast microwells. Cast contains 4 areas containing the inverse for microwell fabrication. B. Agarose is overlaid on the PDMS stamp to generate the microwells. C. Microwells are removed from PDMS stamp, inverted and cut to size (denoted by arrow). D. Cells are added to bottom of imaging chamber slide (denoted by arrow). E. Cut-to-size microwell is overlaid onto cells, trapping cells within the wells. F. Grid is weighed down to prevent movement of microwell or cells escaping. G. Microwell assembly is overlaid mineral oil to prevent evaporation. H. A glass slide is adhered with high vacuum grease to seal the chamber slide. I. Representative DIC image of SmOx cells in 100x100x5 μm wells, imaged with a 20/0.8 NA air lens. (TIF) [file ppat.1010218.s001.tif]

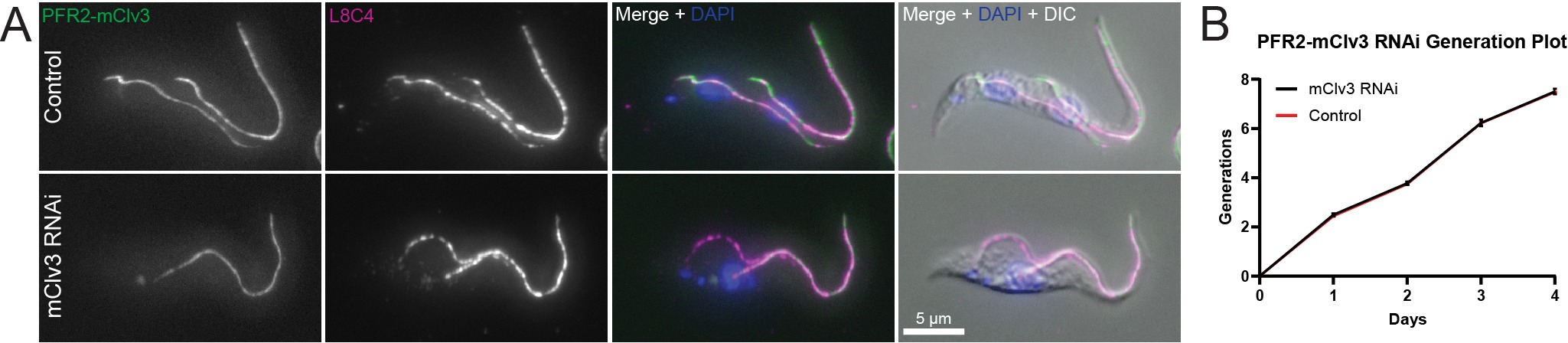

Supplement: S2 Fig — RNAi against the single PFR2-mClv3 allele does not affect cell growth or the paraflagellar rod. A. RNAi against PFR2-mClv3 was induced for 12 h. Cells were harvested and methanol fixed and stained using L8C4 antibody to label the PFR. Native fluorescence of PFR2-mClv3 is shown. Depletion of the PFR2-mClv3 allele through RNAi results in a normal new PFR structure with no PFR2-mClv3 signal. B. RNAi against PFR2-mClv3 was induced for 4 days with 1 μg/ml doxycycline or 70% ethanol as a vehicle control. Cell concentration was counted every 24 h. Graph depicts three independent experiments; error bars depict standard deviation. (TIF) [file ppat.1010218.s002.tif]
